# Best Practices for Using AI Tools as an Author, Peer Reviewer, or Editor

**DOI:** 10.2196/51584

**Published:** 2023-08-31

**Authors:** Tiffany I Leung, Taiane de Azevedo Cardoso, Amaryllis Mavragani, Gunther Eysenbach

**Affiliations:** 1 JMIR Publications, Inc Toronto, ON Canada; 2 Department of Internal Medicine (adjunct) Southern Illinois University School of Medicine Springfield, IL United States; 3 University of Victoria Victoria, BC Canada

**Keywords:** publishing, open access publishing, open science, publication policy, science editing, scholarly publishing, scientific publishing, research, scientific research, editorial, artificial intelligence, AI

## Abstract

The ethics of generative artificial intelligence (AI) use in scientific manuscript content creation has become a serious matter of concern in the scientific publishing community. Generative AI has computationally become capable of elaborating research questions; refining programming code; generating text in scientific language; and generating images, graphics, or figures. However, this technology should be used with caution. In this editorial, we outline the current state of editorial policies on generative AI or chatbot use in authorship, peer review, and editorial processing of scientific and scholarly manuscripts. Additionally, we provide JMIR Publications’ editorial policies on these issues. We further detail JMIR Publications’ approach to the applications of AI in the editorial process for manuscripts in review in a JMIR Publications journal.

## Introduction

Technology tools are useful for making the scientific writing process more timely and effective. Many advances have been made in terms of the tools available to help conduct more sophisticated statistical analysis, manage references, and check grammar. Among these advances, large language model (LLMs) are neural networks trained on large corpora of textual information that can be fine-tuned to respond to natural language queries in a conversational fashion. In late 2022, OpenAI released ChatGPT, an artificial intelligence (AI) chatbot [1] that uses an LLM, which has become enormously popular and a focal point for regulatory debate in a matter of months. Since then, countless LLMs have been developed and launched for research, commercial, and other applications.

The ethics of generative AI use in scientific manuscript content creation has become a serious matter of concern in the scientific publishing community [2,3]. More generally, there are already broader calls for the regulation of AI, and LLMs in particular, in general public use [4,5]. This is because generative AI has computationally become capable of elaborating research questions; refining programming code; generating text in scientific language; and generating images, graphics, or figures. However, this technology should be used with caution. For instance, LLMs may produce errors and misleading information, especially when dealing with technical topics that they may have had limited data to train on. In the technical report released by OpenAI, it is acknowledged that Generative Pre-trained Transformer (GPT)–4 can produce biased and unreliable content [6]. Such biased output can result from inherent biases in the data on which they were trained. A recent study published in the *Journal of Medical Internet Research* showed that ChatGPT was able to generate a highly convincing, fraudulent scientific manuscript article in approximately 1 hour [7]. The authors used tools to detect AI-generated text (AI Detector and AI Text Classifier), and the results were inconclusive, indicating that these tools were unable to determine that the manuscript was generated by ChatGPT. Finally, the authors were able to detect mistakes in the generated article, specifically in the references, as ChatGPT generated fictitious citations. These findings reinforce the importance of having well-established regulations around the use of ChatGPT in the scientific field.

For authors of academic manuscripts, key issues of concern include the need to fact-check AI-generated content of any form (including but not limited to textual information or graphics); assign accountability for AI-generated information; and disclose transparently the use of generative AI in producing any scholarly or scientific work, especially when it impacts the meaning and content of the information submitted for potential publication [8]. For peer reviewers, additional issues pertain to the typical processing of manuscripts, wherein humans traditionally have generated peer review reports and issued editorial decisions on revising, rejecting, or accepting manuscripts. Currently, it is possible to prompt generative AI to facilitate these processes when given specific inputs and prompts as well. For editors, receiving AI-generated material in manuscripts (from authors) or in peer review reports (from peer reviewers) also warrant additional considerations.

In this editorial, we outline the current state of editorial policies on generative AI or chatbot use in authorship, peer review, and editorial processing of scientific and scholarly manuscripts. Additionally, we provide JMIR Publications’ editorial policies on these issues, with the goal of ensuring the integrity of the science published and the publishing process. We further detail JMIR Publications’ approach to the applications of AI in the editorial process for manuscripts in review in a JMIR Publications journal.

## For Authors

In scientific publishing, there is already historical precedent that the transparency of authorship is essential to the integrity of scientific publication [9]. Regarding AI, general consensus already states that AI cannot be a listed coauthor on a manuscript because of the inability for the AI to be accountable for the content written [2,10-13]. The lack of accountability and ability to give consent to be published as a coauthor would be consistent with not listing an AI tool as a coauthor [14]. According to Committee on Publication Ethics (COPE) guidance, “AI tools cannot meet the requirements for authorship as they cannot take responsibility for the submitted work. As non-legal entities, they cannot assert the presence or absence of conflicts of interest nor manage copyright and license agreements” [2]. The World Associate of Medical Editors (WAME) states in their *Recommendations on Chatbots and Generative Artificial Intelligence in Relation to Scholarly Publication* that “Chatbots cannot be authors” [11]. One examination of ChatGPT (the free version of GPT-3) against the Contributor Roles Taxonomy (CRediT) authorship criteria [15] noted that the chatbot meets only 3 of 14 criteria for authorship [16]. Unfortunately, before such widespread publisher policies and recommendations became the norm, some manuscripts and preprints have already been published that identified ChatGPT as a coauthor [13].

At JMIR Publications, early guidance in our knowledge base of editorial policies explained that authors must appropriately include a description of the use of generative AI in the conduct or reporting of scientific work; otherwise, if this information is not a part of the study design (eg, in the Methods section of a manuscript), then providing acknowledgment of the use of generative AI in writing or creating text, figures, or other content for scientific publication is required [17-19]. We welcome authors to submit relevant work to the flagship journal of JMIR Publications, the *Journal of Medical Internet Research*, which now has a section on generative language models (including ChatGPT), where it may be appropriate to submit work that uses such technology as a core component of the work (Table 1). If an author does not use AI to generate any portions of a submitted manuscript, it would be appropriate for the author also to provide a pertinent attestation in their cover letter on submission.

Such acknowledgements must be fully transparent, precise, and complete throughout the submission, editorial, and production processes and will be disclosed upon the publication of a manuscript, if accepted for publication after the disclosure has been provided [19]. In addition, we strongly recommend authors to supply their transcripts, including complete prompts and responses, in supplementary files (whether or not it is published) as exemplified in Eysenbach [20], as this serves as additional information for the peer reviewers or editor to consider in their evaluation of the manuscript.

Authors must also be cautious of the use of generative AI because of its predispositions to hallucination information and references [20-22]. Because generative AI cannot be accountable for the outputs and possible hallucinations that they generate in response to a prompt, authors are accountable for fact- and reference-checking any references suggested by a generative AI tool. Authors must also be cautious of the potential for *unintentional plagiarism* (because the AI may not be able to properly source or cite literature) [23] or overt *AI plagiarism* (the authors passing off or taking credit for the production of statements that were generated by AI). Either form of plagiarism is deemed not acceptable and would be examined carefully in accordance with COPE guidance [24]. Authors may wish to adhere to the WAME recommendation that they “specify what they have done to mitigate the risk of plagiarism, provide a balanced view, and ensure the accuracy of all their references” [11]. Furthermore, instances of suspected or potential scientific misconduct or violations of publication ethics principles, regardless of the involvement or use of generative AI, would be investigated in accordance with JMIR Publications policies, which adhere to COPE guidance.

**Table 1:** Author’s responsibilities when using generative artificial intelligence (AI) in preparing a manuscript.

Guiding principle	Author’s responsibilities
Accountability	Be accountable for the content of AI-generated comments submitted in the manuscript. For example, AI-generated statements should have accompanying citations where appropriate and be fact-checked for accuracy, and generated references should be checked to ensure that they have not been hallucinated. Do not list generative AI as a coauthor.
Transparency	If generative AI was a part of the study design, include appropriate methodological detail in the Methods section of a manuscript. Describe how generative AI was used in the conduct of the scientific work in sufficient detail for a peer-reviewed publication. If generative AI was used to generate manuscript content, then state clearly in the Acknowledgments section how and where generative AI was used. This may include but is not limited to writing or creating text, figures, or other content for scientific publication. Disclose which generative AI tool was used by attesting to its use, such as stating, “I conducted this review with the assistance of [ProductName, Version, from CompanyName, Year].” If no generative AI was used, state in the cover letter of the submission the following: “The author(s) attest that there was no use of generative artificial intelligence (AI) technology in the generation of text, figures, or other informational content of this manuscript.”
Confidentiality	Authors use generative AI at their own risk. Understanding the terms of use of any generative AI is recommended to understand how the content of prompts may be reused by the generative AI and the company that created it.

## For Peer Reviewers

For peer reviewers, JMIR Publications adheres to expectations similar to that for authors: specifically, peer reviewers are accountable for the content of AI-generated comments submitted in a peer review. Consequently, peer reviewers are strongly advised to still ensure that the quality and content of the peer review meet the recommended standards described elsewhere in JMIR Publications policies [25]. However, peer reviewers must remain cautious about the risks of such use, including but not limited to the perpetuation of bias and nonneutral language in AI use (eg, gender, racial, political, or other biases based on individual characteristics) [26,27] and information leakage or breaches of confidentiality [27,28] (Table 2). The latter point on the confidentiality of manuscript information warrants a more extended clarification: when authors agree to open peer review of their JMIR Publications manuscript (ie, on *JMIR Preprints* [29]), information leakage is of lesser concern because authors have already consented to an open peer review process, and their manuscript is publicly viewable. JMIR Publications encourages open peer review [30]. However, in some instances, authors wish to maintain a traditional, closed peer review process; in such cases, peer reviewers may risk information leakage by engaging generative AI in assisting them in the process of peer review report generation.

**Table 2:** Peer reviewer’s responsibilities when using generative artificial intelligence (AI) in peer review.

Guiding principle	Peer reviewer’s responsibilities
Accountability	Be accountable for the content of AI-generated comments submitted in their peer review. The quality and content of the peer review meet the recommended standards in JMIR Publications policies [31].
Transparency	Disclose which generative AI tool was used by attesting to its use at the end of a peer review report (in Comments to Authors), such as stating, “I conducted this review with the assistance of [ProductName, Version, from CompanyName, Year].” Describe in detail how it was used in supporting peer review generation (in Confidential Comments to the Editor). Sufficient detail must be provided so that an editor has a clear and complete understanding of the role of AI in peer review report generation. The handling editor may request the peer reviewer to provide more detail, for example, the prompts used and the responses generated by AI.
Confidentiality	Carefully and thoroughly review the terms of use of any generative AI. If the peer reviewer’s relationship to the content (manuscript) does not adhere to the terms of use, or the peer reviewer doubts that the generative AI maintains the confidentiality of content, do not engage in its use for this task.

In addition to accountability and confidentiality, transparency is essential to ensure the integrity of the peer review process. Agencies such as the US National Institutes of Health (NIH) have issued clear guidance that the use of AI in assisting a review with the grant peer review process is prohibited due to a breach of their confidentiality and nondisclosure agreements [32]. Some publishers have opted to ban generative AI use or restrict use to in-house or licensed technologies [33,34]. The WAME states that “peer reviewers should specify, to authors and each other, any use of chatbots in the evaluation of the manuscript and generation of reviews” [11].

At JMIR Publications, we adhere to this guidance of transparency and disclosure; we do not endorse a ban on generative AI in peer review, which can be counterproductive in various ways [14,35]. Peer reviewers are expected to disclose and describe their use of generative AI (Table 2). As JMIR Publications follows single-blind peer review with unblinding only upon publication, the publisher may include a comment (Editorial Notice) at their discretion, which would accompany the publication history of a manuscript regarding a peer reviewer’s disclosure of AI use during the peer review process. Here, we further elaborate on some of the detailed considerations a peer reviewer must account for when considering generative AI use to support their personal peer review process.

**Figure 1:**
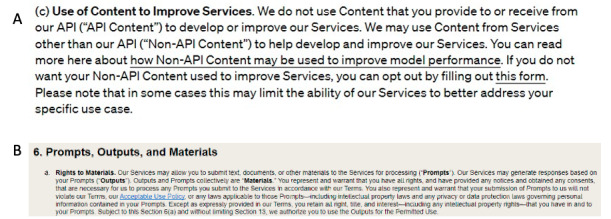
(A) Screenshot of 3(c) from OpenAI’s ChatGPT Terms of Use (Multimedia Appendix 1). (B) Screenshot of 6(a) from Anthropic’s Claude Terms of Service (Multimedia Appendix 2).

Importantly, when peer reviewers use generative AI to support their peer review, they are accountable to ensuring the confidentiality of the peer review process. Detailed and careful review of the terms of use of any generative AI is strongly advised, if not required. Furthermore, if the peer reviewer has any doubts about potential information leakage after a careful review of the terms of use of a generative AI tool, then they should not engage in its use for this task. For example, in the free version of Open AI’s ChatGPT, their March 14, 2023, Terms of Use (Figure 1 and Multimedia Appendix 1) do not exclude the potential for secondary use or reuse of provided information (“Input”), although the use of their application programming interface (API) suggests that they would exclude the reuse of input: “We do not use Content that you provide to or receive from our API to develop or improve our Services. We may use Content from Services other than our API to help develop and improve our Services” [36]. Because there is potential for the input to be reused, JMIR Publications would *not* permit the use of the free version of ChatGPT for assisting with peer review comment generation.

In another example, Anthropic’s Claude also has clearly stated language in their July 8, 2023, Terms of Service (Figure 1 and Multimedia Appendix 2): “You represent and warrant that you have all rights, and have provided any notices and obtained any consents that are necessary for us to process any Prompts you submit to the Services in accordance with our Terms. You also represent and warrant that your submission of Prompts to us will not violate our Terms...including intellectual property laws and any privacy or data protection laws governing personal information contained in your Prompts” [37]. Because peer reviewers do not have “all rights” or have not “obtained any consents” with regard to a manuscript they may review, JMIR Publications would *not* permit the use of the free version of Claude for assisting with peer review comment generation.

Peer reviewers for JMIR Publications journals are advised to carefully review the content of the Peer Reviewer Hub for guidance [25], including guidance on writing a high-quality peer review [31]. Instances of suspected or potential peer review manipulation, fraud, scientific misconduct, or violations of publication ethics principles during the peer review process would be investigated in accordance with JMIR Publications policies, which adhere to COPE guidance.

## For Editors

AI is already in use by some publishers, as an attempt to optimize the editorial workflow. For instance, some publishers have publicly available tools where the authors can add the title, keywords, and abstract of their manuscript, and the AI tool will list the journals that this work is more suitable for. This approach could be time-saving for both the editors and the authors.

Similar to peer reviewers and authors, editors evaluating and issuing decisions about manuscripts are accountable for the content of their decisions and the final decision on the manuscript, whether it is accepted or rejected (Table 3). This includes whether the editor may choose to use generative AI to assist in the summarization of peer review reports or the generation of text for an editorial decision [11,14]. The transparency and maintenance of confidentiality again remain essential, in precisely the same ways as noted for peer reviewers: the editor is accountable for ensuring the confidentiality of the peer review process where it is required (ie, when authors choose not to engage in open peer review).

When editors evaluate peer reviews of a manuscript that they are assigned to, the editor should follow JMIR Publications policies in evaluating the quality, validity, relevance, and professional language use of a peer review. In a recommendation from the WAME, similar to peer reviewers, editors are also accountable for the generated content, the transparency of the disclosure of use, and maintaining confidentiality during the peer review process [11]. Routinely, plagiarism is a serious concern in scientific publishing, and existing tools are able to identify writing that is plagiarized from existing published literature. AI plagiarism occurs when a person generates extensive material using AI and claims it as their own work [7,11,38,39]. Plagiarism detection tools now must encompass AI plagiarism as well [38,40]. To avoid AI plagiarism, authors must disclose the use of generative AI as detailed above. Peer reviews may electively opt to use plagiarism detection tools when performing a peer review and would be required to adhere to appropriate disclosures as previously detailed. Editors (or the publisher) may use tools to detect whether a manuscript presents content written by generative AI, although all users of any AI plagiarism detection tools must again adhere to the principles of transparency and confidentiality. For example, although GPTZero may seem to be a promising option, there is a risk of information leakage or loss of confidentiality, based upon a review of its terms of use [41] (Multimedia Appendix 3). If an editor identifies issues with research integrity regarding any of the above guidance for authors or peer reviewers, then these would be investigated according to JMIR Publications policies.

**Table 3:** Editor’s responsibilities when using generative artificial intelligence (AI) in peer review.

Guiding principle	Editor’s responsibilities
Accountability	Be accountable for the content of their decisions, including AI-generated content, and the final decision on the manuscript, whether it is accepted or rejected. Follow JMIR Publications policies in evaluating the quality, validity, relevance, and professional language use of a peer review. Optionally request peer reviewers who have disclosed generative AI use to provide more detail, for example, the prompts used and the responses generated by AI.
Transparency	Disclose which generative AI tool was used by attesting to its use at the end of a decision, if necessary, such as stating, “I conducted this review with the assistance of [ProductName, Version, from CompanyName, Year].” The publisher may include a comment (Editorial Notice) at their discretion, which would accompany the publication history of a manuscript regarding peer reviewers’ or handling editors’ disclosure of generative AI use during the peer review process.
Confidentiality	Carefully and thoroughly review the terms of use of any generative AI. If the editor’s relationship to the content (manuscript and peer reviews) does not adhere to the terms of use, or the editor doubts that the generative AI maintains the confidentiality of the content, do not engage in its use for this task.

## Closing Comments

The accountability of parties using generative AI, transparency regarding complete disclosure, and the maintenance of confidentiality are fundamental in maintaining the integrity of the scientific record and are key components of JMIR Publications’ editorial policies. Because of the rapidly evolving nature of AI technologies, related policies, regulations [42], investigations [43], and best practices [44,45], JMIR Publications looks forward to continuing to lead and evolve as an innovator in scientific publishing.

## Appendix

Multimedia Appendix 1 OpenAI Terms of Use, updated March 14, 2023.

Multimedia Appendix 2 Anthropic Terms of Service, version 3.0, updated July 8, 2023.

Multimedia Appendix 3 GPTZero Terms of Use, updated January 22, 2023.

## Abbreviations

AI: artificial intelligence
API: application programming interface
ChatGPT: Chat Generative Pre-trained Transformer
COPE: Committee on Publication Ethics
CRediT: Contributor Roles Taxonomy
GPT: Generative Pre-trained Transformer
LLM: large language model
NIH: National Institutes of Health
WAME: World Associate of Medical Editors
