# Supplementary material for: Best Practices for Using AI Tools as an Author, Peer Reviewer, or Editor
Source: J Med Internet Res. 2023 Aug 31;25:e51584. doi: 10.2196/51584 (PMC10502596; doi:10.2196/51584)
Supplement: Multimedia Appendix 1 [file jmir_v25i1e51584_app1.pdf]

Terms of use

Updated
March 14, 2023
Thank you for using OpenAI!

These Terms of Use apply when you use the services of OpenAI, L.L.C. or our affiliates, including our application programming interface, software, tools, developer services, data, documentation, and websites ("Services"). The Terms include our Service Terms, Sharing & Publication Policy, Usage Policies, and other documentation, guidelines, or policies we may provide in writing. By using our Services, you agree to these Terms. Our Privacy Policy explains how we collect and use personal information.

1. Registration and Access

You must be at least 13 years old to use the Services. If you are under 18 you must have your parent or legal guardian's permission to use the Services. If you use the Services on behalf of another person or entity, you must have the authority to accept the Terms on their behalf. You must provide accurate and complete information to register for an account. You may not make your access credentials or account available to others outside your organization, and you are responsible for all activities that occur using your credentials.

2. Usage Requirements

(a) **Use of Services**. You may access, and we grant you a non-exclusive right to use, the Services in accordance with these Terms. You will comply with these Terms and all applicable laws when using the Services. We and our affiliates own all rights, title, and interest in and to the Services.

(b) **Feedback**. We appreciate feedback, comments, ideas, proposals and suggestions for improvements. If you provide any of these things, we may use it without restriction or compensation to you.

(c) **Restrictions**. You may not (i) use the Services in a way that infringes, misappropriates or violates any person's rights; (ii) reverse assemble, reverse compile, decompile, translate or otherwise attempt to discover the source code or underlying components of models, algorithms, and systems of the Services (except to the extent such restrictions are contrary to applicable law); (iii) use output from the Services to develop models that compete with OpenAI; (iv) except as permitted through the API, use any automated or programmatic method to extract data or output from the Services, including scraping, web harvesting, or web data extraction; (v) represent that output from the Services was human-generated when it is not or otherwise violate our Usage Policies; (vi) buy, sell, or transfer API keys without our prior consent; or (vii), send us any personal information of children under 13 or the applicable age of digital consent. You will comply with any rate limits and other requirements in our documentation. You may use Services only in geographies currently supported by OpenAI.

(d) **Third Party Services**. Any third party software, services, or other products you use in connection with the Services are subject to their own terms, and we are not responsible for third party products.

3. Content

(a) **Your Content**. You may provide input to the Services ("Input"), and receive output generated and returned by the Services based on the Input ("Output"). Input and Output are collectively "Content." As between the parties and to the extent permitted by applicable law, you own all Input. Subject to your compliance with these Terms, OpenAI hereby

assigns to you all its right, title and interest in and to Output. This means you can use Content for any purpose, including commercial purposes such as sale or publication, if you comply with these Terms. OpenAI may use Content to provide and maintain the Services, comply with applicable law, and enforce our policies. You are responsible for Content, including for ensuring that it does not violate any applicable law or these Terms.

(b) **Similarity of Content**. Due to the nature of machine learning, Output may not be unique across users and the Services may generate the same or similar output for OpenAI or a third party. For example, you may provide input to a model such as “What color is the sky?” and receive output such as “The sky is blue.” Other users may also ask similar questions and receive the same response. Responses that are requested by and generated for other users are not considered your Content.

(c) **Use of Content to Improve Services**. We do not use Content that you provide to or receive from our API (“API Content”) to develop or improve our Services. We may use Content from Services other than our API (“Non-API Content”) to help develop and improve our Services. You can read more here about how Non-API Content may be used to improve model performance. If you do not want your Non-API Content used to improve Services, you can opt out by filling out this form. Please note that in some cases this may limit the ability of our Services to better address your specific use case.

(d) **Accuracy**. Artificial intelligence and machine learning are rapidly evolving fields of study. We are constantly working to improve our Services to make them more accurate, reliable, safe and beneficial. Given the probabilistic nature of machine learning, use of our Services may in some situations result in incorrect Output that does not accurately reflect real people, places, or facts. You should evaluate the accuracy of any Output as appropriate for your use case, including by using human review of the Output.

4. Fees and Payments

(a) **Fees and Billing**. You will pay all fees charged to your account (“Fees”) according to the prices and terms on the applicable pricing page, or as otherwise agreed between us in writing. We have the right to correct pricing errors or mistakes even if we have already issued an invoice or received payment. You will provide complete and accurate billing information including a valid and authorized payment method. We will charge your payment method on an agreed-upon periodic basis, but may reasonably change the date on which the charge is posted. You authorize OpenAI and its affiliates, and our third-party payment processor(s), to charge your payment method for the Fees. If your payment cannot be completed, we will provide you written notice and may suspend access to the Services until payment is received. Fees are payable in U.S. dollars and are due upon invoice issuance. Payments are nonrefundable except as provided in this Agreement.

(b) **Taxes**. Unless otherwise stated, Fees do not include federal, state, local, and foreign taxes, duties, and other similar assessments (“Taxes”). You are responsible for all Taxes associated with your purchase, excluding Taxes based on our net income, and we may invoice you for such Taxes. You agree to timely pay such Taxes and provide us with documentation showing the payment, or additional evidence that we may reasonably require. OpenAI uses the name and address in your account registration as the place of supply for tax purposes, so you must keep this information accurate and up-to-date.

(c) **Price Changes**. We may change our prices by posting notice to your account and/or to our website. Price increases will be effective 14 days after they are posted, except for increases made for legal reasons or increases made to Beta Services (as defined in our Service Terms), which will be effective immediately. Any price changes will apply to the Fees charged to your account immediately after the effective date of the changes.

(d) **Disputes and Late Payments**. If you want to dispute any Fees or Taxes, please contact ar@openai.com within thirty (30) days of the date of the disputed invoice. Undisputed amounts past due may be subject to a finance charge of 1.5% of the unpaid balance per month. If any amount of your Fees are past due, we may suspend your access to

the Services after we provide you written notice of late payment.

(e) **Free Tier**. You may not create more than one account to benefit from credits provided in the free tier of the Services. If we believe you are not using the free tier in good faith, we may charge you standard fees or stop providing access to the Services.

5. Confidentiality, Security and Data Protection

(a) **Confidentiality**. You may be given access to Confidential Information of OpenAI, its affiliates and other third parties. You may use Confidential Information only as needed to use the Services as permitted under these Terms. You may not disclose Confidential Information to any third party, and you will protect Confidential Information in the same manner that you protect your own confidential information of a similar nature, using at least reasonable care. Confidential Information means nonpublic information that OpenAI or its affiliates or third parties designate as confidential or should reasonably be considered confidential under the circumstances, including software, specifications, and other nonpublic business information. Confidential Information does not include information that: (i) is or becomes generally available to the public through no fault of yours; (ii) you already possess without any confidentiality obligations when you received it under these Terms; (iii) is rightfully disclosed to you by a third party without any confidentiality obligations; or (iv) you independently developed without using Confidential Information. You may disclose Confidential Information when required by law or the valid order of a court or other governmental authority if you give reasonable prior written notice to OpenAI and use reasonable efforts to limit the scope of disclosure, including assisting us with challenging the disclosure requirement, in each case where possible.

(b) **Security**. You must implement reasonable and appropriate measures designed to help secure your access to and use of the Services. If you discover any vulnerabilities or breaches related to your use of the Services, you must promptly contact OpenAI and provide details of the vulnerability or breach.

(c) **Processing of Personal Data**. If you use the Services to process personal data, you must provide legally adequate privacy notices and obtain necessary consents for the processing of such data, and you represent to us that you are processing such data in accordance with applicable law. If you will be using the OpenAI API for the processing of "personal data" as defined in the GDPR or "Personal Information" as defined in CCPA, please fill out this form to request to execute our Data Processing Addendum.

6. Term and Termination

(a) **Termination; Suspension**. These Terms take effect when you first use the Services and remain in effect until terminated. You may terminate these Terms at any time for any reason by discontinuing the use of the Services and Content. We may terminate these Terms for any reason by providing you at least 30 days' advance notice. We may terminate these Terms immediately upon notice to you if you materially breach Sections 2 (Usage Requirements), 5 (Confidentiality, Security and Data Protection), 8 (Dispute Resolution) or 9 (General Terms), if there are changes in relationships with third party technology providers outside of our control, or to comply with law or government requests. We may suspend your access to the Services if you do not comply with these Terms, if your use poses a security risk to us or any third party, or if we suspect that your use is fraudulent or could subject us or any third party to liability.

(b) **Effect on Termination**. Upon termination, you will stop using the Services and you will promptly return or, if instructed by us, destroy any Confidential Information. The sections of these Terms which by their nature should survive termination or expiration should survive, including but not limited to Sections 3 and 5-9.

7. Indemnification; Disclaimer of Warranties; Limitations on Liability

(a) **Indemnity**. You will defend, indemnify, and hold harmless us, our affiliates, and our personnel, from and against any claims, losses, and expenses (including attorneys' fees) arising from or relating to your use of the Services, including your Content, products or services you develop or offer in connection with the Services, and your breach of these Terms or violation of applicable law.

(b) **Disclaimer**. THE SERVICES ARE PROVIDED "AS IS." EXCEPT TO THE EXTENT PROHIBITED BY LAW, WE AND OUR AFFILIATES AND LICENSORS MAKE NO WARRANTIES (EXPRESS, IMPLIED, STATUTORY OR OTHERWISE) WITH RESPECT TO THE SERVICES, AND DISCLAIM ALL WARRANTIES INCLUDING BUT NOT LIMITED TO WARRANTIES OF MERCHANTABILITY, FITNESS FOR A PARTICULAR PURPOSE, SATISFACTORY QUALITY, NON-INFRINGEMENT, AND QUIET ENJOYMENT, AND ANY WARRANTIES ARISING OUT OF ANY COURSE OF DEALING OR TRADE USAGE. WE DO NOT WARRANT THAT THE SERVICES WILL BE UNINTERRUPTED, ACCURATE OR ERROR FREE, OR THAT ANY CONTENT WILL BE SECURE OR NOT LOST OR ALTERED.

(c) **Limitations of Liability**. NEITHER WE NOR ANY OF OUR AFFILIATES OR LICENSORS WILL BE LIABLE FOR ANY INDIRECT, INCIDENTAL, SPECIAL, CONSEQUENTIAL OR EXEMPLARY DAMAGES, INCLUDING DAMAGES FOR LOSS OF PROFITS, GOODWILL, USE, OR DATA OR OTHER LOSSES, EVEN IF WE HAVE BEEN ADVISED OF THE POSSIBILITY OF SUCH DAMAGES. OUR AGGREGATE LIABILITY UNDER THESE TERMS SHALL NOT EXCEED THE GREATER OF THE AMOUNT YOU PAID FOR THE SERVICE THAT GAVE RISE TO THE CLAIM DURING THE 12 MONTHS BEFORE THE LIABILITY AROSE OR ONE HUNDRED DOLLARS ($100). THE LIMITATIONS IN THIS SECTION APPLY ONLY TO THE MAXIMUM EXTENT PERMITTED BY APPLICABLE LAW.

8. Dispute Resolution

YOU AGREE TO THE FOLLOWING MANDATORY ARBITRATION AND CLASS ACTION WAIVER PROVISIONS:

(a) **MANDATORY ARBITRATION**. You and OpenAI agree to resolve any past or present claims relating to these Terms or our Services through final and binding arbitration, except that you have the right to opt out of these arbitration terms, and future changes to these arbitration terms, by filling out this form within 30 days of agreeing to these arbitration terms or the relevant changes.

(b) **Informal Dispute Resolution**. We would like to understand and try to address your concerns prior to formal legal action. Before filing a claim against OpenAI, you agree to try to resolve the dispute informally by sending us notice at dispute-resolution@openai.com of your name, a description of the dispute, and the relief you seek. If we are unable to resolve a dispute within 60 days, you may bring a formal proceeding. Any statute of limitations will be tolled during the 60-day resolution process. If you reside in the EU, the European Commission provides for an online dispute resolution platform, which you can access at https://ec.europa.eu/consumers/odr.

(c) **Arbitration Forum**. Either party may commence binding arbitration through ADR Services, an alternative dispute resolution provider. The parties will pay equal shares of the arbitration fees. If the arbitrator finds that you cannot afford to pay the arbitration fees and cannot obtain a waiver, OpenAI will pay them for you. OpenAI will not seek its attorneys' fees and costs in arbitration unless the arbitrator determines that your claim is frivolous.

(d) **Arbitration Procedures**. The arbitration will be conducted by telephone, based on written submissions, video conference, or in person in San Francisco, California or at another mutually agreed location. The arbitration will be conducted by a sole arbitrator by ADR Services under its then-prevailing rules. All issues are for the arbitrator to decide, except a California court has the authority to determine (i) the scope, enforceability, and arbitrability of this Section 8, including the mass filing procedures below, and (ii) whether you have complied with the pre-arbitration requirements in this section. The amount of any settlement offer will not be disclosed to the arbitrator by either party until after the arbitrator determines the final award, if any.

(e). **Exceptions**. This arbitration section does not require arbitration of the following claims: (i) individual claims brought in small claims court; and (ii) injunctive or other equitable relief to stop unauthorized use or abuse of the Services or intellectual property infringement.

(f) **NO CLASS ACTIONS**. Disputes must be brought on an individual basis only, and may not be brought as a plaintiff or class member in any purported class, consolidated, or representative proceeding. Class arbitrations, class actions,

private attorney general actions, and consolidation with other arbitrations are not allowed. If for any reason a dispute proceeds in court rather than through arbitration, each party knowingly and irrevocably waives any right to trial by jury in any action, proceeding, or counterclaim. This does not prevent either party from participating in a class-wide settlement of claims.

(g) **Mass Filings**. If, at any time, 30 or more similar demands for arbitration are asserted against OpenAI or related parties by the same or coordinated counsel or entities ("Mass Filing"), ADR Services will randomly assign sequential numbers to each of the Mass Filings. Claims numbered 1-10 will be the "Initial Test Cases" and will proceed to arbitration first. The arbitrators will render a final award for the Initial Test Cases within 120 days of the initial pre-hearing conference, unless the claims are resolved in advance or the parties agree to extend the deadline. The parties will then have 90 days (the "Mediation Period") to resolve the remaining cases in mediation based on the awards from the Initial Test Cases. If the parties are unable to resolve the outstanding claims during this time, the parties may choose to opt out of the arbitration process and proceed in court by providing written notice to the other party within 60 days after the Mediation Period. Otherwise, the remaining cases will be arbitrated in their assigned order. Any statute of limitations will be tolled from the time the Initial Test Cases are chosen until your case is chosen as described above.

(h) **Severability**. If any part of this Section 8 is found to be illegal or unenforceable, the remainder will remain in effect, except that if a finding of partial illegality or unenforceability would allow Mass Filing or class or representative arbitration, this Section 8 will be unenforceable in its entirety. Nothing in this section will be deemed to waive or otherwise limit the right to seek public injunctive relief or any other non-waivable right, pending a ruling on the substance of such claim from the arbitrator.

9. General Terms

(a) **Relationship of the Parties**. These Terms do not create a partnership, joint venture or agency relationship between you and OpenAI or any of OpenAI's affiliates. OpenAI and you are independent contractors and neither party will have the power to bind the other or to incur obligations on the other's behalf without the other party's prior written consent.

(b) **Use of Brands**. You may not use OpenAI's or any of its affiliates' names, logos, or trademarks, without our prior written consent.

(c) **U.S. Federal Agency Entities**. The Services were developed solely at private expense and are commercial computer software and related documentation within the meaning of the applicable U.S. Federal Acquisition Regulation and agency supplements thereto.

(d) **Copyright Complaints**. If you believe that your intellectual property rights have been infringed, please send notice to the address below or fill out this form. We may delete or disable content alleged to be infringing and may terminate accounts of repeat infringers.

OpenAI, L.L.C.
3180 18th St
San Francisco, CA 94110
Attn: General Counsel / Copyright Agent

Written claims concerning copyright infringement must include the following information:

- A physical or electronic signature of the person authorized to act on behalf of the owner of the copyright interest;
- A description of the copyrighted work that you claim has been infringed upon;
- A description of where the material that you claim is infringing is located on the site;
- Your address, telephone number, and e-mail address;

A statement by you that you have a good-faith belief that the disputed use is not authorized by the copyright owner, its agent, or the law; and

- A statement by you, made under penalty of perjury, that the above information in your notice is accurate and that you are the copyright owner or authorized to act on the copyright owner's behalf.

(e) **Assignment and Delegation**. You may not assign or delegate any rights or obligations under these Terms, including in connection with a change of control. Any purported assignment and delegation shall be null and void. We may assign these Terms in connection with a merger, acquisition or sale of all or substantially all of our assets, or to any affiliate or as part of a corporate reorganization.

(f) **Modifications**. We may amend these Terms from time to time by posting a revised version on the website, or if an update materially adversely affects your rights or obligations under these Terms we will provide notice to you either by emailing the email associated with your account or providing an in-product notification. Those changes will become effective no sooner than 30 days after we notify you. All other changes will be effective immediately. Your continued use of the Services after any change means you agree to such change.

(g) **Notices**. All notices will be in writing. We may notify you using the registration information you provided or the email address associated with your use of the Services. Service will be deemed given on the date of receipt if delivered by email or on the date sent via courier if delivered by post. OpenAI accepts service of process at this address: OpenAI, L.L.C., 3180 18th Street, San Francisco, CA 94110, Attn: contract-notices@openai.com.

(h) **Waiver and Severability**. If you do not comply with these Terms, and OpenAI does not take action right away, this does not mean OpenAI is giving up any of our rights. Except as provided in Section 8, if any part of these Terms is determined to be invalid or unenforceable by a court of competent jurisdiction, that term will be enforced to the maximum extent permissible and it will not affect the enforceability of any other terms.

(i) **Export Controls**. The Services may not be used in or for the benefit of, exported, or re-exported (a) into any U.S. embargoed countries (collectively, the "Embargoed Countries") or (b) to anyone on the U.S. Treasury Department's list of Specially Designated Nationals, any other restricted party lists (existing now or in the future) identified by the Office of Foreign Asset Control, or the U.S. Department of Commerce Denied Persons List or Entity List, or any other restricted party lists (collectively, "Restricted Party Lists"). You represent and warrant that you are not located in any Embargoed Countries and not on any such restricted party lists. You must comply with all applicable laws related to Embargoed Countries or Restricted Party Lists, including any requirements or obligations to know your end users directly.

(j) **Equitable Remedies**. You acknowledge that if you violate or breach these Terms, it may cause irreparable harm to OpenAI and its affiliates, and OpenAI shall have the right to seek injunctive relief against you in addition to any other legal remedies.

(k) **Entire Agreement**. These Terms and any policies incorporated in these Terms contain the entire agreement between you and OpenAI regarding the use of the Services and, other than any Service specific terms of use or any applicable enterprise agreements, supersedes any prior or contemporaneous agreements, communications, or understandings between you and OpenAI on that subject.

(l) **Jurisdiction, Venue and Choice of Law**. These Terms will be governed by the laws of the State of California, excluding California's conflicts of law rules or principles. Except as provided in the "Dispute Resolution" section, all claims arising out of or relating to these Terms will be brought exclusively in the federal or state courts of San Francisco County, California, USA.

Research
Overview
Index
Product
Overview
ChatGPT
GPT-4
DALL·E 2
Customer stories
Safety standards
API data privacy
Pricing
Safety
Overview
Company
About
Blog
Careers
Charter
Security

OpenAI © 2015–2023 Terms & policiesPrivacy policyBrand guidelines

Twitter
YouTube
GitHub
SoundCloud
LinkedIn
