# Supplementary material for: Best Practices for Using AI Tools as an Author, Peer Reviewer, or Editor
Source: J Med Internet Res. 2023 Aug 31;25:e51584. doi: 10.2196/51584 (PMC10502596; doi:10.2196/51584)
Supplement: Multimedia Appendix 2 [file jmir_v25i1e51584_app2.pdf]

Anthropic Logo

Version 3.0

Effective July 8, 2023

Terms of Service

Our Terms of Service (“**Terms**”) are a legally binding agreement between you and Anthropic, PBC (“**Anthropic**”, “**we**”, or “**us**”). Our Terms govern your access to and use of our website, products, and services, including their web interfaces, application programming interfaces (“**APIs**”), and other software, tools, services, documentation, content, intellectual property, and functionalities that we may make available to you (collectively, our “**Services**”).

Please read our Terms carefully before you use our Services. By accepting our Terms or otherwise accessing or using our Services, you agree to be bound by and comply with our Terms, and acknowledge that you have read and understand our Privacy Policy and Acceptable Use Policy. If you do not agree to our Terms, or if you object to our Privacy Policy or Acceptable Use Policy, you must not access or use our Services.

By accepting our Terms on behalf of a company, organization, or other entity, you represent and warrant that you are authorized to bind the company, organization, or other entity on whose behalf you are accepting our Terms—you and such entity, collectively, are “you” in this case—and you agree on behalf of that entity that such entity is bound by our Terms. If you are accepting our Terms for yourself, then you agree to be personally bound by our Terms—you individually are “you” in this case.

1. Changes to Terms

We may revise and update our Terms from time to time in our sole discretion. If you continue to use the Services after we post the updated Terms on the Services or otherwise give you notice of such changes, it means that you accept and agree to the updated Terms. If you do not accept the updated Terms, you must not continue to access or use the Services.

2. Accounts

To access or use the Services, we may ask you to register for an account and provide certain information to us. Any such information must be correct, current, and complete. You agree that we may use this information to communicate with you about our Services and that our communications to you will satisfy any requirements for legal notices.

You acknowledge that your account is specific to you. You must keep all passwords, API tokens, or other credentials to access or use our Services confidential and not disclose them to any other person or entity. You also must not provide any other person or entity with access to our Services using your account credentials. You are responsible for all activity occurring under your account or with use of your access credentials, and you agree to notify us immediately if you become aware of any unauthorized access to or use of your account or access credentials by sending an email to support@anthropic.com. Unless we specifically state otherwise in a separate agreement with you, we may terminate your access to the Services or your account in accordance with Section 8 below.

You may close your account at any time by contacting us at support@anthropic.com.

3. Evaluation and Additional Services

As applicable, we may permit you to evaluate our Services for a limited time or with limited functionality that may include restrictions on the number of concurrent users, how you may access our Services, or the number of characters per Prompt. Use of our Services for evaluation purposes are for your internal, non-commercial use only.

Your use of certain Services may be subject to your acceptance of additional terms. Such additional terms will supplement our Terms with respect to such Services may revise or update your rights or obligations with respect to those Services, including your obligations to pay fees. If you accept any such terms on behalf of a company, organization, or other entity, you represent and warrant that you are authorized to bind that entity to those additional terms.

4. Use Rights, Requirements, and Restrictions

Please review our Acceptable Use Policy, which outlines the permissible uses of our Services. You may access and use our Services only in compliance with our Terms, our Acceptable Use Policy, and all applicable laws, rules, and regulations (the "**Permitted Use**").

You may not access or use the Services in the following ways, and if any of these restrictions are inconsistent with or ambiguous in relation to the Acceptable Use Policy, the Acceptable Use Policy controls:

- In any manner that violates any applicable law—including, without limitation, any laws about exporting data or software to and from the United States or other countries.
- To develop any products or services that supplant or compete with our Services, including to develop or train any artificial intelligence or machine learning algorithms or models.
- To decompile, reverse engineer, disassemble, or otherwise reduce our Services to human-readable form, except when these restrictions are prohibited by applicable law.
- To crawl, scrape, or otherwise harvest data or information from our Services other than as permitted under these Terms.
- To use our Services or Materials to obtain unauthorized access to any system or information or to deceive any person.
- To infringe, misappropriate, or violate intellectual property or other legal rights (including the rights of publicity or privacy).
- To engage in any other conduct that restricts or inhibits any person from using or enjoying our Services, or that in our sole judgment exposes us—or any of our users, affiliates, or any other third party—to any liability, damages, or detriment of any type, including reputational harms.
- To assist any person in doing any of the above.

5. Ownership of the Services

The Services are owned and operated by us and our affiliates, licensors, and service providers (collectively "**Providers**"). We and our Providers retain all our respective rights, title, and interest, including intellectual property rights, in and to the Services. Other than the rights of access and use expressly granted in our Terms, our Terms do not grant you any right, title, or interest in or to our Services.

6. Prompts, Outputs, and Materials

a. **Rights to Materials.** Our Services may allow you to submit text, documents, or other materials to the Services for processing ("**Prompts**"). Our Services may generate responses based on your Prompts ("**Outputs**"). Outputs and Prompts collectively are "**Materials**." You represent and warrant that you have all rights, and have provided any notices and obtained any consents, that are necessary for us to process any Prompts you submit to the Services in accordance with our Terms. You also represent and warrant that your submission of Prompts to us will not violate our Terms, our Acceptable Use Policy, or any laws applicable to those Prompts—including intellectual property laws and any privacy or data protection laws governing personal information contained in your Prompts. Except as expressly provided in our Terms, you retain all right, title, and interest—including any intellectual property rights—that you have in and to your Prompts. Subject to this Section 6(a) and without limiting Section 13, we authorize you to use the Outputs for the Permitted Use.

b. **Personal Information.** If you submit any personal information to us in connection with your use of the Services, whether as part of your Prompts or otherwise, you acknowledge that our Privacy Policy governs our use and processing of such personal information.

c. **Nature of Outputs.** Responses that the Services generate based on materials submitted by third parties ("**Third-Party Outputs**") may be identical or similar to third-party materials or Outputs that the Services generate based on your Prompts. You acknowledge that Third-Party Outputs are not your Outputs and that you have no right, title, or interest in or to any Third-Party Outputs.

d. **Reliance on Outputs.** We make no representations or warranties with respect to the accuracy of any Outputs. You should not rely on any Outputs without independently confirming their accuracy. Outputs may contain material inaccuracies even if they appear accurate because of their level of detail or specificity. The Services and any Outputs may not reflect correct, current, or complete information.

e. **Our Use of Materials.** We may use Materials to provide, maintain, and improve the Services and to develop other products and services. We will not train our machine learning models on any Prompts or Outputs that are not publicly available; however, if you provide Feedback to us (through the Services or otherwise) regarding any Prompts or Outputs, we may use that Feedback in accordance with Section 11.

7. Viruses

We do not guarantee that our Services will be totally secure or free from bugs or viruses. You are responsible for configuring your information technology, computer programmes and platform in order to access our Services and we recommend that you use your own virus protection software.

You must not misuse the Website or our Services by knowingly introducing viruses, trojans, worms, logic bombs or other material which is malicious or technologically harmful. You must not attempt to gain unauthorized access to the Website, the server on which the Website is stored or any server, computer or database connected to the Website. You must not attack the Website via a denial-of-service attack or a distributed denial-of service attack. By breaching this provision, you would commit a criminal offense under the Computer Misuse Act 1990 (or local law equivalent). We will report any such breach to the relevant law enforcement authorities and we will cooperate with those authorities by disclosing your identity to them. In the event of such a breach, your right to use the Website will cease immediately.

8. Modification, Suspension, Discontinuation of the Services

Our Services are novel and will evolve. Unless we specifically state otherwise in a separate agreement with you, we reserve the right to temporarily or permanently modify, suspend, or discontinue the Services or your access to the Services or account at any time, in our sole discretion, without notice to you, and we will not be liable for any change to or any suspension or discontinuation of the Services or your access to them, to the maximum extent permissible under applicable law.

9. Fees and Payment

You may be required to pay fees to us to access or use the Services or certain features of the Services. You are responsible for paying any applicable fees listed on the Services. Except as expressly provided in these Terms, all fees are non-refundable, to the fullest extent permissible under applicable law.

Fees may be recurring or based on usage. If these fees are specified to be recurring or based on usage, you agree that we may charge such fees on a periodic basis to the payment method you specify at the time of your initial purchase (your “**Payment Method**”). By using a Payment Method to pay fees, you are expressly agreeing that we are authorized to charge to the Payment Method the fees, together with any applicable taxes.

Except where we specifically agree otherwise in a separate contract with you, you acknowledge and agree that any fees for access to or use of the Services may increase at any time. Additional fees may apply for additional Services or features of the Services that Anthropic may make available. In those cases, we will provide you with notice before charging the additional fees. If we charge additional fees in connection with our Services, you will have an opportunity to review and accept the additional fees that you will be charged, before being charged. If you do not accept any such additional fees, we may discontinue your access to the Services or features. You acknowledge and agree that we will not be held liable for any errors caused by third-party payment processors that we may use.

10. Third-Party Services and Links

Our Services may use or be used in connection with third-party content, services, or integrations. We do not control or accept responsibility for any loss or damage that may arise from your use of any third-party content, services, and integrations, for which we make no representations or warranties. Your use of any third-party content, services, and integrations is at your own risk and subject to any terms, conditions, or policies (including privacy policies) applicable to such third-party content, services, and integrations.

11. Feedback

You grant to us an irrevocable, royalty-free, perpetual license to use all feedback, ideas, or suggested improvements you provide to us (through the Services or otherwise) regarding the Services, Prompts, or Outputs (collectively “**Feedback**”), and you agree that we and our Providers may use the Feedback--together with the related Prompts and Outputs or any derivative thereof--in any manner without any payment or credit to you, including in connection with our development, improvement, and marketing of our Services or other products or services.

12. Confidentiality

a. **Confidential Information.** In connection with your use of the Services, we may provide information that we identify as confidential or proprietary or that should reasonably be considered confidential or proprietary under the circumstances (“**Confidential Information**”).

b. **Obligations.** You may only use our Confidential Information in connection with the Permitted Use. You may only share our Confidential Information with your employees and agents who have a need to know such Confidential Information for the Permitted Use and who are bound by obligations of confidentiality at least as protective as those provided in our Terms. You will protect the Confidential Information from unauthorized use, access, or disclosure at least as diligently as you protect your own highly confidential information, and with no less than reasonable care. You are responsible for all uses and disclosures of Confidential Information by anyone to whom you provide access to such Confidential Information (your "**Representatives**").

c. **Exclusions.** Your obligations under this section do not apply to Confidential Information that you can demonstrate (i) was already known to you without confidentiality obligations at the time of disclosure by us; (ii) was disclosed to you by a third party without a duty of confidentiality; (iii) was or becomes publicly available through no fault of yours or of your Representatives; or (iv) was independently developed by you without use of the Confidential Information. You may disclose the Confidential Information to the extent such disclosure is required by applicable law, but you agree that you will, except where expressly prohibited by applicable law, notify us of any such required disclosure promptly and fully cooperate with us in seeking to limit such disclosure.

d. **Destruction.** You will destroy all Confidential Information in your and your Representatives' possession or control promptly upon our request.

13. Disclaimer of Warranties, Limitations of Liability, and Indemnity

a. YOUR USE OF THE SERVICES AND MATERIALS IS SOLELY AT YOUR OWN RISK. THE SERVICES AND OUTPUTS ARE PROVIDED ON AN "AS IS" AND "AS AVAILABLE" BASIS AND, TO THE FULLEST EXTENT PERMISSIBLE UNDER APPLICABLE LAW, ARE PROVIDED WITHOUT WARRANTIES OF ANY KIND, WHETHER EXPRESS, IMPLIED, OR STATUTORY. WE AND OUR PROVIDERS EXPRESSLY DISCLAIM ANY AND ALL WARRANTIES OF FITNESS FOR A PARTICULAR PURPOSE, TITLE, MERCHANTABILITY, ACCURACY, AVAILABILITY, RELIABILITY, SECURITY, PRIVACY, COMPATIBILITY, NON-INFRINGEMENT, AND ANY WARRANTY IMPLIED BY COURSE OF DEALING, COURSE OF PERFORMANCE, OR TRADE USAGE.

b. TO THE FULLEST EXTENT PERMISSIBLE UNDER APPLICABLE LAW, IN NO EVENT WILL WE, OUR PROVIDERS, OR OUR OR THEIR RESPECTIVE AFFILIATES, INVESTORS, DIRECTORS, OFFICERS, EMPLOYEES, AGENTS, SUCCESSORS OR ASSIGNS (COLLECTIVELY, THE "**ANTHROPIC PARTIES**"), BE LIABLE FOR ANY DIRECT, INDIRECT, PUNITIVE, INCIDENTAL, SPECIAL, CONSEQUENTIAL, EXEMPLARY, OR OTHER DAMAGES ARISING OUT OF OR IN ANY WAY RELATED TO THE SERVICES, THE MATERIALS, OR THESE TERMS, WHETHER BASED IN CONTRACT, TORT (INCLUDING NEGLIGENCE), STRICT LIABILITY, OR OTHER THEORY, EVEN IF ANY ANTHROPIC PARTIES HAVE BEEN ADVISED OF THE POSSIBILITY OF DAMAGES, AND EVEN IF THE DAMAGES ARE FORESEEABLE.

c. TO THE FULLEST EXTENT PERMISSIBLE UNDER APPLICABLE LAW, THE ANTHROPIC PARTIES' TOTAL AGGREGATE LIABILITY TO YOU FOR ALL DAMAGES, LOSSES AND CAUSES OF ACTION ARISING OUT OF OR IN ANY WAY RELATED TO THE SERVICES, THE MATERIALS, OR THESE TERMS, WHETHER IN CONTRACT, TORT (INCLUDING NEGLIGENCE) OR OTHERWISE, WILL NOT EXCEED THE GREATER OF THE AMOUNT YOU PAID TO US FOR ACCESS TO OR USE OF THE SERVICES (IF ANY) IN THE SIX MONTHS PRECEDING THE DATE SUCH DAMAGES, LOSSES, AND CAUSES OF ACTION FIRST AROSE AND $100. THE FOREGOING LIMITATIONS ARE ESSENTIAL TO THESE TERMS AND WE WOULD NOT OFFER THE SERVICES TO YOU UNDER THESE TERMS WITHOUT THESE LIMITATIONS.

d. YOU AGREE TO INDEMNIFY AND HOLD HARMLESS THE ANTHROPIC PARTIES FROM AND AGAINST ANY AND ALL LIABILITIES, CLAIMS, DAMAGES, EXPENSES (INCLUDING REASONABLE ATTORNEYS'

FEES AND COSTS), AND OTHER LOSSES ARISING OUT OF OR RELATED TO YOUR BREACH OR ALLEGED BREACH OF THESE TERMS; YOUR ACCESS TO, USE OF, OR ALLEGED USE OF THE SERVICES OR THE MATERIALS; YOUR FEEDBACK; ANY PRODUCTS OR SERVICES THAT YOU DEVELOP, OFFER, OR OTHERWISE MAKE AVAILABLE USING OR OTHERWISE IN CONNECTION WITH THE SERVICES; YOUR VIOLATION OF APPLICABLE LAW OR ANY THIRD-PARTY RIGHT; AND ANY ACTUAL OR ALLEGED FRAUD, INTENTIONAL MISCONDUCT, GROSS NEGLIGENCE, OR CRIMINAL ACTS COMMITTED BY YOU OR YOUR EMPLOYEES OR AGENTS. WE RESERVE THE RIGHT TO ENGAGE SEPARATE COUNSEL AND PARTICIPATE IN OR ASSUME THE EXCLUSIVE DEFENSE AND CONTROL OF ANY MATTER OTHERWISE SUBJECT TO INDEMNIFICATION BY YOU HEREUNDER, IN WHICH CASE YOU AGREE TO COOPERATE WITH US AND SUCH SEPARATE COUNSEL AS WE REASONABLY REQUEST.

e. THE LAWS OF SOME JURISDICTIONS DO NOT ALLOW THE DISCLAIMER OF IMPLIED WARRANTIES OR CERTAIN TYPES OF DAMAGES, SO SOME OR ALL OF THE DISCLAIMERS AND LIMITATIONS OF LIABILITY IN THESE TERMS MAY NOT APPLY TO YOU.

14. Termination

Unless we specifically state otherwise in a separate agreement with you, we may terminate our Terms at any time by notice to you, and our Terms will terminate automatically without notice upon your violation or breach of any provisions of our Terms. You may also terminate our Terms at any time, for any reason, by discontinuing your access to and use of the Services. Upon termination, the rights granted to you under our Terms to access and use the Services will immediately terminate, and you must destroy all Confidential Information in your or your Representatives' possession or control. Upon termination of our Terms or your access to the Services, we may at our option delete any Materials or other data associated with your account. Sections 5, 6, 9 (with respect to fees outstanding as of such expiration or termination), and 10 through 16 will survive any expiration or termination of our Terms.

15. Arbitration

PLEASE READ THIS SECTION CAREFULLY BECAUSE IT AFFECTS YOUR RIGHTS. BY AGREEING TO BINDING ARBITRATION, YOU WAIVE YOUR RIGHT TO LITIGATE DISPUTES THROUGH A COURT AND TO HAVE A JUDGE OR JURY DECIDE YOUR CASE. THE LAWS OF SOME JURISDICTIONS DO NOT ALLOW MANDATORY ARBITRATION PROVISIONS OR CLASS ACTION WAIVERS, SO SOME OR ALL OF THIS SECTION 15 MAY NOT APPLY TO YOU.

In order to expedite and control the cost of disputes, Anthropic and you agree that any legal or equitable claim, dispute, action, or proceeding arising from or related to your use of the Services or these Terms ("**Dispute**") will be resolved as follows to the fullest extent permitted by applicable law. This applies to all Disputes, whether based in contract, tort, statute, fraud, misrepresentation, or any other legal theory, even if the Dispute arises after the termination of these Terms. YOU UNDERSTAND AND AGREE THAT YOU AND ANTHROPIC ARE HEREBY WAIVING THE RIGHT TO A TRIAL BY JURY AND THE RIGHT TO JOIN AND PARTICIPATE IN A CLASS ACTION, TO THE FULLEST EXTENT PERMITTED UNDER THE LAW.

a. **Opt-Out of Arbitration Agreement.** If you are an individual consumer, you can opt out of arbitration within 30 days of the date that you first agreed to these Terms (including any earlier version). If you are an individual consumer and have previously agreed to arbitration, then you may opt out of any future revisions to the arbitration provision within 30 days of receiving notice of the updated arbitration provision, in which case the prior version of the arbitration provision will apply. To opt out of arbitration (or revisions to this arbitration provision), you must send your name, residence address, username, email or phone number you use for your Services account, and a clear statement that you want to opt out of this arbitration agreement (or of the revisions to it), and you must send them here: **notices@anthropic.com**.

b. **Notice of Dispute.** In the event of a Dispute, you or Anthropic must give the other a written statement that sets forth the name, address, and contact information of the party giving it, the facts giving rise to the Dispute, and a proposed solution (a "**Notice of Dispute**"). You must send any Notice of Dispute by first class U.S. Mail to Anthropic at 548 Market Street, PMB 90375, San Francisco, CA 94104-5401 and also via email to **notices@anthropic.com**. Anthropic will send any Notice of Dispute to you by first class U.S. Mail to your address if Anthropic has it, or otherwise to your email address. You and Anthropic will attempt to resolve any Dispute through informal negotiation within 45 days from the date the Notice of Dispute is sent. After 45 days, you or Anthropic may commence arbitration. An arbitrator will decide any disputes over whether this subsection has been violated, and has the power to enjoin the filing or prosecution of arbitrations. Unless prohibited by applicable law, the arbitrator will not administer any arbitration unless the requirements of this subsection have been met.

c. **Mediation, Binding Arbitration, and Governing Law.** You and Anthropic will endeavor to settle any Dispute by mediation under the Mediation Rules of Judicial Arbitration and Mediation Services, Inc. ("**JAMS**"). The place of mediation will be San Francisco, California. Any Dispute which has not been resolved by mediation as provided herein within 30 days after appointment of a mediator or such time period as you or Anthropic may otherwise agree, will be finally resolved by binding arbitration as described in this Section 15. You are giving up the right to litigate (or participate in as a party or class member) all Disputes in court before a judge or jury. Instead, all Disputes will be resolved before a neutral arbitrator, whose decision will be final except for a limited right of appeal under the Federal Arbitration Act. The arbitrator will decide all issues pertaining to arbitrability, including his or her own jurisdictional validity and enforceability of the Agreement (e.g., unconscionability). For the avoidance of doubt, this is not meant to reduce any powers granted to the arbitrator under the applicable JAMS rules. The place of arbitration will be San Francisco, California. Any court with jurisdiction over the parties may enforce the arbitrator's award.

d. **Class Action Waiver.** TO THE FULLEST EXTENT PERMISSIBLE UNDER APPLICABLE LAW, YOU AND ANTHROPIC AGREE THAT ANY PROCEEDINGS TO RESOLVE OR LITIGATE ANY DISPUTE IN ANY FORUM WILL BE CONDUCTED SOLELY ON AN INDIVIDUAL BASIS, AND NEITHER YOU NOR ANTHROPIC WILL SEEK TO HAVE ANY DISPUTE HEARD AS A CLASS ACTION OR IN ANY OTHER PROCEEDING IN WHICH EITHER PARTY ACTS OR PROPOSES TO ACT IN A REPRESENTATIVE CAPACITY. No arbitration or proceeding will be combined with another without the prior written consent of all parties to all affected arbitrations or proceedings. CLASS ACTIONS AND CLASS ARBITRATIONS ARE NOT PERMITTED; for example, you may bring a claim only on your own behalf and cannot seek relief that would affect other Services users. Nor may an arbitrator consolidate arbitrations unless all parties agree. If there is a final judicial determination that the limitations of this paragraph are unenforceable as to a particular claim or a particular request for relief (such as a request for injunctive relief), then the parties agree that such a claim or request for relief will be decided by a court after all other claims and requests for relief are arbitrated.

e. **Mass Arbitrations.** If 10 or more claimants submit similar Notices of Dispute or file similar arbitrations and are represented by the same or coordinated counsel, all of the cases must be resolved in arbitration in stages using staged bellwether proceedings. You agree to do this even though the resolution of some claims might be delayed. In the first stage, the parties will select up to 5 cases to be filed in arbitration and resolved by separate arbitrators. In the meantime, no other cases may be filed in arbitration. Nor may the arbitration provider accept, administer or demand payment for fees for other arbitrations. If the remaining cases are not settled after the first stage is done, the parties will repeat the process. These staged bellwether proceedings will continue until all cases are resolved. If this subsection applies to a Notice of Dispute, any statute of limitations applicable to the listed claims will be tolled from the time the first cases are selected for bellwether proceedings until the claimant's Notice of Dispute is selected for a bellwether proceeding or otherwise resolved. A court will have the authority to enforce this subsection, including the power to enjoin the filing or prosecution of arbitrations or assessment of related fees.

f. **Arbitration Procedures.** Any arbitration will be conducted by JAMS under the JAMS Comprehensive Arbitration Rules and Procedures ("**JAMS Rules**") in effect at the time the Dispute is filed. You may request a telephonic or in-person hearing by following the JAMS Rules. In a dispute involving $10,000 or less, any hearing will be telephonic unless the arbitrator finds good cause to hold an in-person hearing instead. To the

extent the forum provided by JAMS is unavailable, Anthropic and you agree to select a mutually agreeable alternative dispute resolution service and that such alternative dispute resolution service will apply the JAMS Rules. Subject to the limitations of liability contained herein, the arbitrator may award the same damages to you individually as a court could. The arbitrator may award declaratory or injunctive relief only to you individually, and only to the extent required to satisfy your individual claim.

g. **Arbitration Fees.** Whoever files the arbitration will pay the initial filing fee. If Anthropic files, then Anthropic will pay; if you file, then you will pay unless you get a fee waiver under the applicable arbitration rules. Each party will bear the expense of that party's attorneys, experts, and witnesses, and other expenses, regardless of which party prevails, but a party may recover any or all expenses (including attorney's fees) from another party if the arbitrator, applying applicable law, so determines.

h. **Filing Period.** TO THE FULLEST EXTENT PERMISSIBLE UNDER APPLICABLE LAW, ANY DISPUTE UNDER THESE TERMS MUST BE FILED WITHIN ONE (1) YEAR IN AN ARBITRATION PROCEEDING. The one-year period begins on the earliest date when any of the events giving rise to the Dispute first occurs. If a claim is not submitted within one year, it is permanently barred. This period can only be extended by the written consent of both parties. No statutes or provisions of law that would toll or otherwise affect the time in which a party may bring a claim will operate to extend the period limited in this Section 15, and any such statutes and provisions are hereby waived, to the fullest extent permissible under applicable law.

i. **Enforceability.** If the waiver of class actions above is found unenforceable, or this entire section is found unenforceable, then this entire section will be null and void. If that happens, you and Anthropic agree that the section below on exclusive jurisdiction and governing law will govern any Dispute.

j. **ALL ANTHROPIC PARTIES ARE INTENDED THIRD-PARTY BENEFICIARIES OF THE ARBITRATION CLAUSES IN THIS SECTION 15.**

16. Miscellaneous

a. **Additional Terms.** When using our Services, you agree to comply with and are subject to any guidelines, rules, or supplemental terms applicable to such Services that may be posted on the Services from time to time. To the extent that we ask you to review and accept any supplemental terms that expressly conflict with our Terms, the supplemental terms will supersede our Terms with respect to your use of the portion of the Services governed by such supplemental terms, solely to the extent of the conflict.

b. **Entire Agreement; No Assignment.** Our Terms and any other terms expressly incorporated by reference form the entire agreement between you and us regarding the subject matter of our Terms. Our Terms may not be transferred or assigned by you without our prior written consent, but may be assigned by us without restriction.

c. **Equitable Relief.** You agree that (a) no adequate remedy exists at law if you breach Section 4 (Use Rights, Requirements, and Restrictions), Section 12 (Confidentiality), or Section 15 (Arbitration); (b) it would be difficult to determine the damages resulting from such breach, and any such breach would cause irreparable harm; and (c) a grant of injunctive relief provides the best remedy for any such breach. Therefore, you waive any opposition to such injunctive relief, as well as any demand that we prove actual damage or post a bond or other security in connection with such injunctive relief.

d. **No Joint Venture, Partnership, Employment, or Agency Relationship.** You agree that no joint venture, partnership, employment, or agency relationship exists between you and us because of our Terms or your access to or use of the Services.

e. **No Sponsorship.** You may not, without our prior written consent, use our name, logo, or other trademarks to promote products or services other than the Services, or in any other way that implies our affiliation, endorsement, or sponsorship.

f. **Severability.** If any provision of our Terms is held by a court or other tribunal of competent jurisdiction to be invalid, illegal, or unenforceable for any reason, such provision will be eliminated or limited to the minimum extent necessary to comply with applicable law, such that the remaining provisions of our Terms will continue in full force and effect.

g. **No Waiver.** No waiver by us of any term or condition set forth in our Terms will be deemed a further or continuing waiver of such term or condition, or a waiver of any other term or condition, and any failure by us to assert a right or provision under our Terms will not constitute a waiver of such right or provision.

h. **Legal Compliance.** We may comply with governmental, court, and law enforcement requests or requirements relating to provision or use of the Services, or to information provided to or collected under our Terms. We reserve the right, at our sole discretion, to report Prompts or Outputs to law enforcement.

i. **Governing Law; Exclusive Jurisdiction.** Our Terms will be governed by, and construed and interpreted in accordance with, the laws of the State of California without giving effect to conflict of law principles. If a lawsuit or court proceeding is permitted under our Terms notwithstanding Section 15, you and Anthropic agree that any such dispute will be litigated in the state or federal courts located in San Francisco, California, and you and Anthropic submit to the personal and exclusive jurisdiction of those courts. By using the Services, you waive any claims that may arise under the laws of other jurisdictions.

j. **U.S. Government Use.** The Services were developed solely at private expense and are commercial computer software and commercial computer software documentation within the meaning of the applicable Federal Acquisition Regulations and their agency supplements. Accordingly, U.S. Government users of the Services will have only those rights that are granted to all other end users of the Services pursuant to these Terms.

k. **Export and Sanctions.** You may not export or provide access to the Services into any U.S. embargoed countries or to anyone on (i) the U.S. Treasury Department’s list of Specially Designated Nationals, (ii) any other restricted party lists identified by the Office of Foreign Asset Control, (iii) the U.S. Department of Commerce Denied Persons List or Entity List, or (iv) any other restricted party lists. You represent and warrant that you and anyone accessing or using the Services on your behalf, or using your account credentials, are not such persons or entities and are not located in any such country.
