# Supplementary material for: Best Practices for Using AI Tools as an Author, Peer Reviewer, or Editor
Source: J Med Internet Res. 2023 Aug 31;25:e51584. doi: 10.2196/51584 (PMC10502596; doi:10.2196/51584)
Supplement: Multimedia Appendix 3 [file jmir_v25i1e51584_app3.pdf]

TERMS OF USE

Last updated January 22, 2023

TABLE OF CONTENTS

1. AGREEMENT TO TERMS
2. INTELLECTUAL PROPERTY RIGHTS
3. USER REPRESENTATIONS
4. USER REGISTRATION
5. FEES AND PAYMENT
6. FREE TRIAL
7. CANCELLATION
8. PROHIBITED ACTIVITIES
9. USER GENERATED CONTRIBUTIONS
10. CONTRIBUTION LICENSE
11. SOCIAL MEDIA
12. SUBMISSIONS
13. U.S. GOVERNMENT RIGHTS
14. SITE MANAGEMENT
15. PRIVACY POLICY
16. TERM AND TERMINATION
17. MODIFICATIONS AND INTERRUPTIONS
18. GOVERNING LAW
19. DISPUTE RESOLUTION
20. CORRECTIONS
21. DISCLAIMER
22. LIMITATIONS OF LIABILITY
23. INDEMNIFICATION
24. USER DATA
25. ELECTRONIC COMMUNICATIONS, TRANSACTIONS, AND SIGNATURES
26. CALIFORNIA USERS AND RESIDENTS
27. MISCELLANEOUS
28. CONTACT US

1. AGREEMENT TO TERMS

These Terms of Use constitute a legally binding agreement made between you, whether personally or on behalf of an entity

(“you”) and GPTZero Inc. (" **Company** ", “**we**”, “**us**”, or “**our**”), concerning your access to and use of the https://gptzero.me website as well as any other media form, media channel, mobile website or mobile application related, linked, or otherwise connected thereto (collectively, the “Site”). We are registered in New Jersey , United States and have our registered office at 6800 Frist Center , Princeton , NJ 08544 . You agree that by accessing the Site, you have read, understood, and agree to be bound by all of these Terms of Use . IF YOU DO NOT AGREE WITH ALL OF THESE TERMS OF USE , THEN YOU ARE EXPRESSLY PROHIBITED FROM USING THE SITE AND YOU MUST DISCONTINUE USE IMMEDIATELY.

Supplemental terms and conditions or documents that may be posted on the Site from time to time are hereby expressly incorporated herein by reference. We reserve the right, in our sole discretion, to make changes or modifications to these Terms of Use from time to time. We will alert you about any changes by updating the “Last updated” date of these Terms of Use , and you waive any right to receive specific notice of each such change. Please ensure that you check the applicable Terms every time you use our Site so that you understand which Terms apply. You will be subject to, and will be deemed to have been made aware of and to have accepted, the changes in any revised Terms of Use by your continued use of the Site after the date such revised Terms of Use are posted.

The information provided on the Site is not intended for distribution to or use by any person or entity in any jurisdiction or country where such distribution or use would be contrary to law or regulation or which would subject us to any registration requirement within such jurisdiction or country. Accordingly, those persons who choose to access the Site from other locations do so on their own initiative and are solely responsible for compliance with local laws, if and to the extent local laws are applicable.

The Site is not tailored to comply with industry-specific regulations (Health Insurance Portability and Accountability Act (HIPAA), Federal Information Security Management Act (FISMA), etc.), so if your interactions would be subjected to such laws, you may not use this Site. You may not use the Site in a way that would violate the Gramm-Leach-Bliley Act (GLBA).

The Site is intended for users who are at least 18 years old. Persons under the age of 18 are not permitted to use or register for the Site.

2. INTELLECTUAL PROPERTY RIGHTS

Unless otherwise indicated, the Site is our proprietary property and all source code, databases, functionality, software, website designs, audio, video, text, photographs, and graphics on the Site (collectively, the “Content”) and the trademarks, service marks, and logos contained therein (the “Marks”) are owned or controlled by us or licensed to us, and are protected by copyright and trademark laws and various other intellectual property rights and unfair competition laws of the United States, international copyright laws, and international conventions. The Content and the Marks are provided on the Site “AS IS” for your information and personal use only. Except as expressly provided in these Terms of Use , no part of the Site and no Content or Marks may be copied, reproduced, aggregated, republished, uploaded, posted, publicly displayed, encoded, translated, transmitted, distributed, sold, licensed, or otherwise exploited for any commercial purpose whatsoever, without our express prior written permission.

Provided that you are eligible to use the Site, you are granted a limited license to access and use the Site and to download or print a copy of any portion of the Content to which you have properly gained access solely for your personal, non-commercial use. We reserve all rights not expressly granted to you in and to the Site, the Content and the Marks.

3. USER REPRESENTATIONS

By using the Site, you represent and warrant that: (1) all registration information you submit will be true, accurate, current, and complete; (2) you will maintain the accuracy of such information and promptly update such registration information as necessary; (3) you have the legal capacity and you agree to comply with these Terms of Use ; (4) you are not a minor in the jurisdiction in which you reside ; (5) you will not access the Site through automated or non-human means, whether through a bot, script or otherwise; (6) you will not use the Site for any illegal or unauthorized purpose; and (7) your use of the Site will not violate any applicable law or regulation.

If you provide any information that is untrue, inaccurate, not current, or incomplete, we have the right to suspend or terminate your account and refuse any and all current or future use of the Site (or any portion thereof).

4. USER REGISTRATION

You may be required to register with the Site. You agree to keep your password confidential and will be responsible for all use of your account and password. We reserve the right to remove, reclaim, or change a username you select if we determine, in our sole discretion, that such username is inappropriate, obscene, or otherwise objectionable.

5. FEES AND PAYMENT

We accept the following forms of payment:

- Visa
- Mastercard
- American Express
- Discover
- PayPal

You may be required to purchase or pay a fee to access some of our services. You agree to provide current, complete, and accurate purchase and account information for all purchases made via the Site. You further agree to promptly update account and payment information, including email address, payment method, and payment card expiration date, so that we can complete your transactions and contact you as needed. We bill you through an online billing account for purchases made via the Site. Sales tax will be added to the price of purchases as deemed required by us. We may change prices at any time. All payments shall be in U.S. dollars.

You agree to pay all charges or fees at the prices then in effect for your purchases, and you authorize us to charge your chosen payment provider for any such amounts upon making your purchase. If your purchase is subject to recurring charges, then you consent to our charging your payment method on a recurring basis without requiring your prior approval for each recurring charge, until you notify us of your cancellation.

We reserve the right to correct any errors or mistakes in pricing, even if we have already requested or received payment. We also reserve the right to refuse any order placed through the Site.

6. FREE TRIAL

We offer a 30-day free trial to new users who register with the Site. The account will be charged according to the user's chosen subscription at the end of the free trial.

7. CANCELLATION

All purchases are non-refundable. You can cancel your subscription at any time by logging into your account . Your cancellation will take effect at the end of the current paid term.

If you are unsatisfied with our services, please email us at team@gptzero.me .

8. PROHIBITED ACTIVITIES

You may not access or use the Site for any purpose other than that for which we make the Site available. The Site may not be used in connection with any commercial endeavors except those that are specifically endorsed or approved by us.

As a user of the Site, you agree not to:

- Systematically retrieve data or other content from the Site to create or compile, directly or indirectly, a collection, compilation, database, or directory without written permission from us.
- Trick, defraud, or mislead us and other users, especially in any attempt to learn sensitive account information such as user passwords.
- Circumvent, disable, or otherwise interfere with security-related features of the Site, including features that prevent or restrict the use or copying of any Content or enforce limitations on the use of the Site and/or the Content contained therein.
- Disparage, tarnish, or otherwise harm, in our opinion, us and/or the Site.
- Use any information obtained from the Site in order to harass, abuse, or harm another person.
- Make improper use of our support services or submit false reports of abuse or misconduct.
- Use the Site in a manner inconsistent with any applicable laws or regulations.
- Engage in unauthorized framing of or linking to the Site.
- Upload or transmit (or attempt to upload or to transmit) viruses, Trojan horses, or other material, including excessive use of capital letters and spamming (continuous posting of repetitive text), that interferes with any party's uninterrupted use and enjoyment of the Site or modifies, impairs, disrupts, alters, or interferes with the use, features, functions, operation, or maintenance of the Site.
- Engage in any automated use of the system, such as using scripts to send comments or messages, or using any data mining, robots, or similar data gathering and extraction tools.
- Delete the copyright or other proprietary rights notice from any Content.
- Attempt to impersonate another user or person or use the username of another user.
- Upload or transmit (or attempt to upload or to transmit) any material that acts as a passive or active information collection or transmission mechanism, including without limitation, clear graphics interchange formats ("gifs"), 1×1 pixels, web bugs, cookies, or other similar devices (sometimes referred to as "spyware" or "passive collection mechanisms" or "pcms").
- Interfere with, disrupt, or create an undue burden on the Site or the networks or services connected to the Site.
- Harass, annoy, intimidate, or threaten any of our employees or agents engaged in providing any portion of the Site to

you.
- Attempt to bypass any measures of the Site designed to prevent or restrict access to the Site, or any portion of the Site.
- Copy or adapt the Site's software, including but not limited to Flash, PHP, HTML, JavaScript, or other code.
- Except as permitted by applicable law, decipher, decompile, disassemble, or reverse engineer any of the software comprising or in any way making up a part of the Site.
- Except as may be the result of standard search engine or Internet browser usage, use, launch, develop, or distribute any automated system, including without limitation, any spider, robot, cheat utility, scraper, or offline reader that accesses the Site, or using or launching any unauthorized script or other software.
- Use a buying agent or purchasing agent to make purchases on the Site.
- Make any unauthorized use of the Site, including collecting usernames and/or email addresses of users by electronic or other means for the purpose of sending unsolicited email, or creating user accounts by automated means or under false pretenses.
- Use the Site as part of any effort to compete with us or otherwise use the Site and/or the Content for any revenue-generating endeavor or commercial enterprise.
- Sell or otherwise transfer your profile.
- Use the Site to advertise or offer to sell goods and services.

9. USER GENERATED CONTRIBUTIONS

The Site does not offer users to submit or post content. We may provide you with the opportunity to create, submit, post, display, transmit, perform, publish, distribute, or broadcast content and materials to us or on the Site, including but not limited to text, writings, video, audio, photographs, graphics, comments, suggestions, or personal information or other material (collectively, "Contributions"). Contributions may be viewable by other users of the Site and through third-party websites. As such, any Contributions you transmit may be treated in accordance with the Site Privacy Policy. When you create or make available any Contributions, you thereby represent and warrant that:

- The creation, distribution, transmission, public display, or performance, and the accessing, downloading, or copying of your Contributions do not and will not infringe the proprietary rights, including but not limited to the copyright, patent, trademark, trade secret, or moral rights of any third party.
- You are the creator and owner of or have the necessary licenses, rights, consents, releases, and permissions to use and to authorize us, the Site, and other users of the Site to use your Contributions in any manner contemplated by the Site and these Terms of Use .
- You have the written consent, release, and/or permission of each and every identifiable individual person in your Contributions to use the name or likeness of each and every such identifiable individual person to enable inclusion and use of your Contributions in any manner contemplated by the Site and these Terms of Use .
- Your Contributions are not false, inaccurate, or misleading.
- Your Contributions are not unsolicited or unauthorized advertising, promotional materials, pyramid schemes, chain letters, spam, mass mailings, or other forms of solicitation.
- Your Contributions are not obscene, lewd, lascivious, filthy, violent, harassing, libelous, slanderous, or otherwise objectionable (as determined by us).
- Your Contributions do not ridicule, mock, disparage, intimidate, or abuse anyone.
- Your Contributions are not used to harass or threaten (in the legal sense of those terms) any other person and to promote violence against a specific person or class of people.

- Your Contributions do not violate any applicable law, regulation, or rule.
- Your Contributions do not violate the privacy or publicity rights of any third party.
- Your Contributions do not violate any applicable law concerning child pornography, or otherwise intended to protect the health or well-being of minors.
- Your Contributions do not include any offensive comments that are connected to race, national origin, gender, sexual preference, or physical handicap.
- Your Contributions do not otherwise violate, or link to material that violates, any provision of these Terms of Use , or any applicable law or regulation.

Any use of the Site in violation of the foregoing violates these Terms of Use and may result in, among other things, termination or suspension of your rights to use the Site.

10. CONTRIBUTION LICENSE

You and the Site agree that we may access, store, process, and use any information and personal data that you provide following the terms of the Privacy Policy and your choices (including settings).

By submitting suggestions or other feedback regarding the Site, you agree that we can use and share such feedback for any purpose without compensation to you.

We do not assert any ownership over your Contributions. You retain full ownership of all of your Contributions and any intellectual property rights or other proprietary rights associated with your Contributions. We are not liable for any statements or representations in your Contributions provided by you in any area on the Site. You are solely responsible for your Contributions to the Site and you expressly agree to exonerate us from any and all responsibility and to refrain from any legal action against us regarding your Contributions.

11. SOCIAL MEDIA

As part of the functionality of the Site, you may link your account with online accounts you have with third-party service providers (each such account, a “Third-Party Account”) by either: (1) providing your Third-Party Account login information through the Site; or (2) allowing us to access your Third-Party Account, as is permitted under the applicable terms and conditions that govern your use of each Third-Party Account. You represent and warrant that you are entitled to disclose your Third-Party Account login information to us and/or grant us access to your Third-Party Account, without breach by you of any of the terms and conditions that govern your use of the applicable Third-Party Account, and without obligating us to pay any fees or making us subject to any usage limitations imposed by the third-party service provider of the Third-Party Account. By granting us access to any Third-Party Accounts, you understand that (1) we may access, make available, and store (if applicable) any content that you have provided to and stored in your Third-Party Account (the “Social Network Content”) so that it is available on and through the Site via your account, including without limitation any friend lists and (2) we may submit to and receive from your Third-Party Account additional information to the extent you are notified when you link your account with the Third-Party Account. Depending on the Third-Party Accounts you choose and subject to the privacy settings that you have set in such Third-Party Accounts, personally identifiable information that you post to your Third-Party Accounts may be available on and through your account on the Site. Please note that if a Third-Party Account or associated service becomes unavailable or our access to such Third-Party Account is terminated by the third-party service provider, then Social Network Content may no longer be available on and through the Site. You will have the ability to

disable the connection between your account on the Site and your Third-Party Accounts at any time. PLEASE NOTE THAT YOUR RELATIONSHIP WITH THE THIRD-PARTY SERVICE PROVIDERS ASSOCIATED WITH YOUR THIRD-PARTY ACCOUNTS IS GOVERNED SOLELY BY YOUR AGREEMENT(S) WITH SUCH THIRD-PARTY SERVICE PROVIDERS. We make no effort to review any Social Network Content for any purpose, including but not limited to, for accuracy, legality, or non-infringement, and we are not responsible for any Social Network Content. You acknowledge and agree that we may access your email address book associated with a Third-Party Account and your contacts list stored on your mobile device or tablet computer solely for purposes of identifying and informing you of those contacts who have also registered to use the Site. You can deactivate the connection between the Site and your Third-Party Account by contacting us using the contact information below or through your account settings (if applicable). We will attempt to delete any information stored on our servers that was obtained through such Third-Party Account, except the username and profile picture that become associated with your account.

12. SUBMISSIONS

You acknowledge and agree that any questions, comments, suggestions, ideas, feedback, or other information regarding the Site ("Submissions") provided by you to us are non-confidential and shall become our sole property. We shall own exclusive rights, including all intellectual property rights, and shall be entitled to the unrestricted use and dissemination of these Submissions for any lawful purpose, commercial or otherwise, without acknowledgment or compensation to you. You hereby waive all moral rights to any such Submissions, and you hereby warrant that any such Submissions are original with you or that you have the right to submit such Submissions. You agree there shall be no recourse against us for any alleged or actual infringement or misappropriation of any proprietary right in your Submissions.

13. U.S. GOVERNMENT RIGHTS

Our services are “commercial items” as defined in Federal Acquisition Regulation (“FAR”) 2.101. If our services are acquired by or on behalf of any agency not within the Department of Defense (“DOD”), our services are subject to the terms of these Terms of Use in accordance with FAR 12.212 (for computer software) and FAR 12.211 (for technical data). If our services are acquired by or on behalf of any agency within the Department of Defense, our services are subject to the terms of these Terms of Use in accordance with Defense Federal Acquisition Regulation (“DFARS”) 227.7202- 3. In addition, DFARS 252.227-7015 applies to technical data acquired by the DOD. This U.S. Government Rights clause is in lieu of, and supersedes, any other FAR, DFARS, or other clause or provision that addresses government rights in computer software or technical data under these Terms of Use .

14. SITE MANAGEMENT

We reserve the right, but not the obligation, to: (1) monitor the Site for violations of these Terms of Use ; (2) take appropriate legal action against anyone who, in our sole discretion, violates the law or these Terms of Use , including without limitation, reporting such user to law enforcement authorities; (3) in our sole discretion and without limitation, refuse, restrict access to, limit the availability of, or disable (to the extent technologically feasible) any of your Contributions or any portion thereof; (4) in our sole discretion and without limitation, notice, or liability, to remove from the Site or otherwise disable all files and content that are excessive in size or are in any way burdensome to our systems; and (5) otherwise manage the Site in a manner designed to protect our rights and property and to facilitate the proper functioning of the Site.

15. PRIVACY POLICY

We care about data privacy and security. Please review our Privacy Policy: **gptzero.me/privacy-policy.html** . By using the Site, you agree to be bound by our Privacy Policy, which is incorporated into these Terms of Use . Please be advised the Site is hosted in the United States . If you access the Site from any other region of the world with laws or other requirements governing personal data collection, use, or disclosure that differ from applicable laws in the United States , then through your continued use of the Site, you are transferring your data to the United States , and you agree to have your data transferred to and processed in the United States .

16. TERM AND TERMINATION

These Terms of Use shall remain in full force and effect while you use the Site. WITHOUT LIMITING ANY OTHER PROVISION OF THESE TERMS OF USE , WE RESERVE THE RIGHT TO, IN OUR SOLE DISCRETION AND WITHOUT NOTICE OR LIABILITY, DENY ACCESS TO AND USE OF THE SITE (INCLUDING BLOCKING CERTAIN IP ADDRESSES), TO ANY PERSON FOR ANY REASON OR FOR NO REASON, INCLUDING WITHOUT LIMITATION FOR BREACH OF ANY REPRESENTATION, WARRANTY, OR COVENANT CONTAINED IN THESE TERMS OF USE OR OF ANY APPLICABLE LAW OR REGULATION. WE MAY TERMINATE YOUR USE OR PARTICIPATION IN THE SITE OR DELETE YOUR ACCOUNT AND ANY CONTENT OR INFORMATION THAT YOU POSTED AT ANY TIME, WITHOUT WARNING, IN OUR SOLE DISCRETION.

If we terminate or suspend your account for any reason, you are prohibited from registering and creating a new account under your name, a fake or borrowed name, or the name of any third party, even if you may be acting on behalf of the third party. In addition to terminating or suspending your account, we reserve the right to take appropriate legal action, including without limitation pursuing civil, criminal, and injunctive redress.

17. MODIFICATIONS AND INTERRUPTIONS

We reserve the right to change, modify, or remove the contents of the Site at any time or for any reason at our sole discretion without notice. However, we have no obligation to update any information on our Site. We also reserve the right to modify or discontinue all or part of the Site without notice at any time. We will not be liable to you or any third party for any modification, price change, suspension, or discontinuance of the Site.

We cannot guarantee the Site will be available at all times. We may experience hardware, software, or other problems or need to perform maintenance related to the Site, resulting in interruptions, delays, or errors. We reserve the right to change, revise, update, suspend, discontinue, or otherwise modify the Site at any time or for any reason without notice to you. You agree that we have no liability whatsoever for any loss, damage, or inconvenience caused by your inability to access or use the Site during any downtime or discontinuance of the Site. Nothing in these Terms of Use will be construed to obligate us to maintain and support the Site or to supply any corrections, updates, or releases in connection therewith.

18. GOVERNING LAW

These Terms of Use and your use of the Site are governed by and construed in accordance with the laws of the State of Delaware applicable to agreements made and to be entirely performed within the State of Delaware , without regard to its conflict of law principles.

19. DISPUTE RESOLUTION

Informal Negotiations

To expedite resolution and control the cost of any dispute, controversy, or claim related to these Terms of Use (each "Dispute" and collectively, the “Disputes”) brought by either you or us (individually, a “Party” and collectively, the “Parties”), the Parties agree to first attempt to negotiate any Dispute (except those Disputes expressly provided below) informally for at least thirty (30) days before initiating arbitration. Such informal negotiations commence upon written notice from one Party to the other Party.

Binding Arbitration

If the Parties are unable to resolve a Dispute through informal negotiations, the Dispute (except those Disputes expressly excluded below) will be finally and exclusively resolved by binding arbitration. YOU UNDERSTAND THAT WITHOUT THIS PROVISION, YOU WOULD HAVE THE RIGHT TO SUE IN COURT AND HAVE A JURY TRIAL. The arbitration shall be commenced and conducted under the Commercial Arbitration Rules of the American Arbitration Association ("AAA") and, where appropriate, the AAA’s Supplementary Procedures for Consumer Related Disputes ("AAA Consumer Rules"), both of which are available at the AAA website www.adr.org. Your arbitration fees and your share of arbitrator compensation shall be governed by the AAA Consumer Rules and, where appropriate, limited by the AAA Consumer Rules. If such costs are determined by the arbitrator to be excessive, we will pay all arbitration fees and expenses. The arbitration may be conducted in person, through the submission of documents, by phone, or online. The arbitrator will make a decision in writing, but need not provide a statement of reasons unless requested by either Party. The arbitrator must follow applicable law, and any award may be challenged if the arbitrator fails to do so. Except where otherwise required by the applicable AAA rules or applicable law, the arbitration will take place in Delaware . Except as otherwise provided herein, the Parties may litigate in court to compel arbitration, stay proceedings pending arbitration, or to confirm, modify, vacate, or enter judgment on the award entered by the arbitrator.

If for any reason, a Dispute proceeds in court rather than arbitration, the Dispute shall be commenced or prosecuted in the state and federal courts located in Delaware , and the Parties hereby consent to, and waive all defenses of lack of personal jurisdiction, and forum non conveniens with respect to venue and jurisdiction in such state and federal courts . Application of the United Nations Convention on Contracts for the International Sale of Goods and the Uniform Computer Information Transaction Act (UCITA) are excluded from these Terms of Use .

In no event shall any Dispute brought by either Party related in any way to the Site be commenced more than one (1) years after the cause of action arose. If this provision is found to be illegal or unenforceable, then neither Party will elect to arbitrate any Dispute falling within that portion of this provision found to be illegal or unenforceable and such Dispute shall be decided by a court of competent jurisdiction within the courts listed for jurisdiction above, and the Parties agree to submit to the personal jurisdiction of that court.

Restrictions

The Parties agree that any arbitration shall be limited to the Dispute between the Parties individually. To the full extent permitted by law, (a) no arbitration shall be joined with any other proceeding; (b) there is no right or authority for any Dispute to be arbitrated on a class-action basis or to utilize class action procedures; and (c) there is no right or authority for any Dispute to be brought in a purported representative capacity on behalf of the general public or any other persons.

Exceptions to Informal Negotiations and Arbitration

The Parties agree that the following Disputes are not subject to the above provisions concerning informal negotiations and binding arbitration: (a) any Disputes seeking to enforce or protect, or concerning the validity of, any of the intellectual property rights of a Party; (b) any Dispute related to, or arising from, allegations of theft, piracy, invasion of privacy, or unauthorized use; and (c) any claim for injunctive relief. If this provision is found to be illegal or unenforceable, then neither Party will elect to arbitrate any Dispute falling within that portion of this provision found to be illegal or unenforceable and such Dispute shall be decided by a court of competent jurisdiction within the courts listed for jurisdiction above, and the Parties agree to submit to the personal jurisdiction of that court.

20. CORRECTIONS

There may be information on the Site that contains typographical errors, inaccuracies, or omissions, including descriptions, pricing, availability, and various other information. We reserve the right to correct any errors, inaccuracies, or omissions and to change or update the information on the Site at any time, without prior notice.

21. DISCLAIMER

THE SITE IS PROVIDED ON AN AS-IS AND AS-AVAILABLE BASIS. YOU AGREE THAT YOUR USE OF THE SITE AND OUR SERVICES WILL BE AT YOUR SOLE RISK. TO THE FULLEST EXTENT PERMITTED BY LAW, WE DISCLAIM ALL WARRANTIES, EXPRESS OR IMPLIED, IN CONNECTION WITH THE SITE AND YOUR USE THEREOF, INCLUDING, WITHOUT LIMITATION, THE IMPLIED WARRANTIES OF MERCHANTABILITY, FITNESS FOR A PARTICULAR PURPOSE, AND NON-INFRINGEMENT. WE MAKE NO WARRANTIES OR REPRESENTATIONS ABOUT THE ACCURACY OR COMPLETENESS OF THE SITE'S CONTENT OR THE CONTENT OF ANY WEBSITES LINKED TO THE SITE AND WE WILL ASSUME NO LIABILITY OR RESPONSIBILITY FOR ANY (1) ERRORS, MISTAKES, OR INACCURACIES OF CONTENT AND MATERIALS, (2) PERSONAL INJURY OR PROPERTY DAMAGE, OF ANY NATURE WHATSOEVER, RESULTING FROM YOUR ACCESS TO AND USE OF THE SITE, (3) ANY UNAUTHORIZED ACCESS TO OR USE OF OUR SECURE SERVERS AND/OR ANY AND ALL PERSONAL INFORMATION AND/OR FINANCIAL INFORMATION STORED THEREIN, (4) ANY INTERRUPTION OR CESSATION OF TRANSMISSION TO OR FROM THE SITE, (5) ANY BUGS, VIRUSES, TROJAN HORSES, OR THE LIKE WHICH MAY BE TRANSMITTED TO OR THROUGH THE SITE BY ANY THIRD PARTY, AND/OR (6) ANY ERRORS OR OMISSIONS IN ANY CONTENT AND MATERIALS OR FOR ANY LOSS OR DAMAGE OF ANY KIND INCURRED AS A RESULT OF THE USE OF ANY CONTENT POSTED, TRANSMITTED, OR OTHERWISE MADE AVAILABLE VIA THE SITE. WE DO NOT WARRANT, ENDORSE, GUARANTEE, OR ASSUME RESPONSIBILITY FOR ANY PRODUCT OR SERVICE ADVERTISED OR OFFERED BY A THIRD PARTY THROUGH THE SITE, ANY HYPERLINKED WEBSITE, OR ANY WEBSITE OR MOBILE APPLICATION FEATURED IN ANY BANNER OR OTHER ADVERTISING, AND WE WILL NOT BE A PARTY TO OR IN ANY WAY BE RESPONSIBLE FOR MONITORING ANY TRANSACTION BETWEEN YOU AND ANY THIRD-PARTY PROVIDERS OF PRODUCTS OR SERVICES. AS WITH THE PURCHASE OF A PRODUCT OR SERVICE THROUGH ANY MEDIUM OR IN ANY ENVIRONMENT, YOU SHOULD USE YOUR BEST JUDGMENT AND EXERCISE CAUTION WHERE

APPROPRIATE.

22. LIMITATIONS OF LIABILITY

IN NO EVENT WILL WE OR OUR DIRECTORS, EMPLOYEES, OR AGENTS BE LIABLE TO YOU OR ANY THIRD PARTY FOR ANY DIRECT, INDIRECT, CONSEQUENTIAL, EXEMPLARY, INCIDENTAL, SPECIAL, OR PUNITIVE DAMAGES, INCLUDING LOST PROFIT, LOST REVENUE, LOSS OF DATA, OR OTHER DAMAGES ARISING FROM YOUR USE OF THE SITE, EVEN IF WE HAVE BEEN ADVISED OF THE POSSIBILITY OF SUCH DAMAGES. NOTWITHSTANDING ANYTHING TO THE CONTRARY CONTAINED HEREIN, OUR LIABILITY TO YOU FOR ANY CAUSE WHATSOEVER AND REGARDLESS OF THE FORM OF THE ACTION, WILL AT ALL TIMES BE LIMITED TO THE AMOUNT PAID, IF ANY, BY YOU TO US DURING THE SIX (6) MONTH PERIOD PRIOR TO ANY CAUSE OF ACTION ARISING . CERTAIN US STATE LAWS AND INTERNATIONAL LAWS DO NOT ALLOW LIMITATIONS ON IMPLIED WARRANTIES OR THE EXCLUSION OR LIMITATION OF CERTAIN DAMAGES. IF THESE LAWS APPLY TO YOU, SOME OR ALL OF THE ABOVE DISCLAIMERS OR LIMITATIONS MAY NOT APPLY TO YOU, AND YOU MAY HAVE ADDITIONAL RIGHTS.

23. INDEMNIFICATION

You agree to defend, indemnify, and hold us harmless, including our subsidiaries, affiliates, and all of our respective officers, agents, partners, and employees, from and against any loss, damage, liability, claim, or demand, including reasonable attorneys’ fees and expenses, made by any third party due to or arising out of: (1) use of the Site; (2) breach of these Terms of Use ; (3) any breach of your representations and warranties set forth in these Terms of Use ; (4) your violation of the rights of a third party, including but not limited to intellectual property rights; or (5) any overt harmful act toward any other user of the Site with whom you connected via the Site. Notwithstanding the foregoing, we reserve the right, at your expense, to assume the exclusive defense and control of any matter for which you are required to indemnify us, and you agree to cooperate, at your expense, with our defense of such claims. We will use reasonable efforts to notify you of any such claim, action, or proceeding which is subject to this indemnification upon becoming aware of it.

24. USER DATA

We will maintain certain data that you transmit to the Site for the purpose of managing the performance of the Site, as well as data relating to your use of the Site. Although we perform regular routine backups of data, you are solely responsible for all data that you transmit or that relates to any activity you have undertaken using the Site. You agree that we shall have no liability to you for any loss or corruption of any such data, and you hereby waive any right of action against us arising from any such loss or corruption of such data.

25. ELECTRONIC COMMUNICATIONS, TRANSACTIONS, AND SIGNATURES

Visiting the Site, sending us emails, and completing online forms constitute electronic communications. You consent to receive electronic communications, and you agree that all agreements, notices, disclosures, and other communications we provide to you electronically, via email and on the Site, satisfy any legal requirement that such communication be in writing.

YOU HEREBY AGREE TO THE USE OF ELECTRONIC SIGNATURES, CONTRACTS, ORDERS, AND OTHER RECORDS, AND TO ELECTRONIC DELIVERY OF NOTICES, POLICIES, AND RECORDS OF TRANSACTIONS INITIATED OR COMPLETED BY US OR VIA THE SITE. You hereby waive any rights or requirements under any statutes, regulations, rules, ordinances, or other laws in any jurisdiction which require an original signature or delivery or retention of non-electronic records, or to payments or the granting of credits by any means other than electronic means.

26. CALIFORNIA USERS AND RESIDENTS

If any complaint with us is not satisfactorily resolved, you can contact the Complaint Assistance Unit of the Division of Consumer Services of the California Department of Consumer Affairs in writing at 1625 North Market Blvd., Suite N 112, Sacramento, California 95834 or by telephone at (800) 952-5210 or (916) 445-1254.

27. MISCELLANEOUS

These Terms of Use and any policies or operating rules posted by us on the Site or in respect to the Site constitute the entire agreement and understanding between you and us. Our failure to exercise or enforce any right or provision of these Terms of Use shall not operate as a waiver of such right or provision. These Terms of Use operate to the fullest extent permissible by law. We may assign any or all of our rights and obligations to others at any time. We shall not be responsible or liable for any loss, damage, delay, or failure to act caused by any cause beyond our reasonable control. If any provision or part of a provision of these Terms of Use is determined to be unlawful, void, or unenforceable, that provision or part of the provision is deemed severable from these Terms of Use and does not affect the validity and enforceability of any remaining provisions. There is no joint venture, partnership, employment or agency relationship created between you and us as a result of these Terms of Use or use of the Site. You agree that these Terms of Use will not be construed against us by virtue of having drafted them. You hereby waive any and all defenses you may have based on the electronic form of these Terms of Use and the lack of signing by the parties hereto to execute these Terms of Use .

28. CONTACT US

In order to resolve a complaint regarding the Site or to receive further information regarding use of the Site, please contact us at:

GPTZero Inc.
35 Olden St
Princeton , NJ 08544
United States
team@gptzero.me
